# Editorial: Advances in Autoimmune Myasthenia Gravis

**DOI:** 10.3389/fimmu.2020.01688

**Published:** 2020-08-28

**Authors:** Anna Rostedt Punga, Linda Kusner, Sonia Berrih-Aknin, Rozen Le Panse

**Affiliations:** ^1^Department of Neuroscience, Clinical Neurophysiology, Uppsala University, Uppsala, Sweden; ^2^Department of Pharmacology and Physiology, The George Washington University, Washington, DC, United States; ^3^Sorbonne University, INSERM, Association Institute of Myology, Center of Research in Myology, Paris, France

**Keywords:** autoantibodies, CD4 T cells, thymus, therapy, miRNA

Myasthenia gravis (MG) is an autoimmune neuromuscular disorder characterized by impaired neuromuscular transmission, which causes fluctuating fatigable muscle weakness. MG is a prototypical autoimmune disease with well-defined autoantibodies that target the neuromuscular junction. The majority of MG patients have autoantibodies against the acetylcholine receptor (AChR) and a smaller proportion of patients have autoantibodies against the muscle-specific tyrosine kinase (MuSK) or the low-density lipoprotein-related protein 4 (LRP4) (Borges and Richman) (1). Furthermore, several other antigenic targets, such as agrin, Kv1.4 potassium channel, rapsyn, cortactin, acetylcholinesterase (AChE), collagen Q (ColQ), and collagen XIII, have been reported. Although the pathogenicity and specificity of these autoantibodies for MG have not been fully characterized, their presence could help in better understanding the variability in disease severity. Moreover, they could provide a diagnostic/prognostic value for the management of MG patients (Fichtner et al.; Lazaridis and Tzartos).

The existence of B cells, which produce autoantibodies, is dependent on the interaction with CD4^+^ T cells—both are key factors in MG. Thymic regulatory T cells are not efficiently suppressive, and T helper cells are resistant to suppression (2). Phenotypic variation of regulatory T cells and functional impairment are more pronounced in the thymus than in peripheral cells (Truffault et al.). Thymic epithelial cells from MG patients appear to play a central role in CD4^+^ T cell defect *via* the release of soluble factors, such as TSLP (Thymic Stromal LymphoPoietin) (Truffault et al.). T helper (Th) 1, Th2, Th17, and T follicular helper (Tfh) cells are involved in MG pathogenic mechanisms. An increase in the levels of interleukin (IL)-21, IL-4, IL-10, and IL-17A is observed in CD4^+^ T peripheral cells in AChR antibody seropositive (AChR^+^) MG, as compared to healthy controls (Çebi et al.). Among CD4^+^ T cells, the percentage of Th17 cells is increased in AChR+ MG patients. ICOS (Inducible T-cell COStimulator) and PD-1 (Programmed cell death protein 1), two molecules associated with Tfh cell function, are also highly expressed on CD4^+^CXCR5^+^ Tfh cells in AChR+ MG. Tfh cells can be stratified in Tfh regulatory cells, or Tfh1, Tfh2, and Tfh17 cells that differentially affect B-cell differentiation. In AChR+ MG patients, the percentage of peripheral Tfh17 cells (CXCR3^−^CCR6^+^CD4^+^ T cells) is also increased but Tfh1 and Tfh2 cells remain unaffected. Some of these changes are also observed to a lesser degree in AChR- MG patients (Çebi et al.) and in MuSK antibody seropositive (MuSK^+^) MG patients (3). Immunosuppressive treatments commonly used in MG do not affect these cells but enhance IL-10 in CD4 T cells (Çebi et al.), and also B cells (4), suggesting a role of IL-10 in favoring immuno-regulatory mechanisms.

Classical treatments of MG include chronic treatments such as AChE inhibitors and general immunosuppressive drugs (5), thymectomy (6), as well as acute treatment for deterioration such as intravenous immunoglobulin and plasmapheresis (5). A new line of molecules is now available and is used for refractory MG. One of these is Rituximab, an anti-CD20 B-cell depleting monoclonal antibody which is often used as a second-line of treatment in combination with conventional immunosuppressants. Rituximab seems more efficient in MuSK^+^ MG patients, with the reduction in MuSK antibodies being associated with clinical improvement (Marino et al.). Other therapies aiming at targeting B cells are also emerging (Huda).

Most recently, medications inhibiting the cleavage of the complement protein C5 have been evaluated in clinical trials. Eculizumab (a monoclonal antibody) has obtained authorization from the US Food and Drug Administration to be used in MG. A second generation of C5 inhibitor, Zilucoplan (a macrocyclic), has also recently entered clinical trials (Albazli et al.). Molecules blocking the function of FcRn are of interest for autoimmune diseases. Inhibition of FcRn reduces the ability to recycle IgGs and thereby removes them from circulation. Agents such as Efgartigimod (IgG1 Fc fragment) or Nicocalimab, Rozanolixizumab, and RCT-140 (monoclonal antibodies) are in clinical trials for MG (Gable and Guptill).

Other therapeutic approaches are still at the preclinical phase and have demonstrated beneficial effects on experimental MG (EAMG) rodent models. If oral or nasal administration of AChR fragments suppress autoimmunity in EAMG (Yamada et al.) (7), the recombinant extracellular domain of MuSK may also be effective in inducing oral tolerance in MuSK^+^ EAMG (Reuveni et al.). Oral tolerance is a phenomenon based on suppressing immune responses in the gut where microbiota could play a role. In that way, probiotics could balance the gut microbiota and have beneficial effects in EAMG (Rinaldi et al.).

Even though MG has been studied for a number of years, the understanding of the etiological mechanisms is still evolving. In AChR^+^ MG, the thymus is known to play a central role in disease onset either in the early- onset form of the disease or in MG-associated thymoma (8). The analyses of enriched pathways from “omics” data might reveal new unexplored pathways central in MG development (Cron et al.; Yamada et al.). It is well-known that genetic predispositions exist in MG patients (9), however, additional epigenetic changes occur. The expression of small non-coding RNA, microRNA (miRNA), is dysregulated in the thymus of AChR^+^ MG patients (10, 11) and could be involved in thymic changes associated with MG, linked to thymic inflammation and ectopic germinal center development (Bortone et al.; Cron et al.). Circulating miRNAs are also potential biomarkers since they are differentially expressed in the serum of MG patients (Sabre et al.). Specific circulating miRNAs have been associated with AChR^+^ and MuSK^+^ MG subtypes and their expression is regulated by treatment with immunosuppression and thymectomy (Fiorillo et al.; Sabre et al.). Investigations into the triggering events that lead to MG are still needed. Sexual hormones can affect, for example miRNAs, and in addition, favor autoimmunity in women (Fiorillo et al.) (12). Environmental factors are also candidates for driving/perpetuating autoimmunity, such as pathogen infection, endocrinal disruptors, and microbiota changes.

Although MG is a relatively well-characterized autoimmune disease, recent studies shed light on the mechanisms of development of this pathology and, most importantly, make it possible to propose more effective tools for monitoring and more effective treatments with fewer side effects. This Research Topic, dedicated to autoimmune MG, addresses these different aspects, both with Original Research articles and Reviews of the literature.

## Author Contributions

AP, LK, SB-A, and RL wrote the editorial. All authors contributed to the article and approved the submitted version.

## Conflict of Interest

The authors declare that the research was conducted in the absence of any commercial or financial relationships that could be construed as a potential conflict of interest.
